# Advances in mechanical ventilation over the past 25 years: from protective ventilation to post-ICU recovery

**DOI:** 10.31744/einstein_journal/2026Suppl_5ED0001

**Published:** 2026-09-15

**Authors:** Caroline Gomes Mól, Carmen Silvia Valente Barbas, Bruno Franco Mazza, Raquel Afonso Caserta Eid, Thais Dias Midega

**Affiliations:** 1 Hospital Israelita Albert Einstein São Paulo SP Brazil Hospital Israelita Albert Einstein, São Paulo, SP, Brazil.; 2 Faculdade Israelita de Ciências da Saúde Albert Einstein São Paulo SP Brazil Faculdade Israelita de Ciências da Saúde Albert Einstein, São Paulo, SP, Brazil.

Over the past 25 years, the definition of success in mechanical ventilation has progressively changed. In the early era of protective ventilation, success was measured almost exclusively by survival through the acute phase of respiratory failure. Today, growing evidence on post-ICU functional and cognitive outcomes has broadened this definition, extending it to the quality of life patients regain after critical illness.

Twenty-five years ago, the ARDSNet trial marked the beginning of the protective ventilation era. Limiting tidal volume to 6 mL/kg of predicted body weight, with a plateau pressure ≤30cmH_2_O, reduced mortality in patients with acute lung injury and acute respiratory distress syndrome (ARDS), compared to higher-volume strategy.^(1)^ This landmark not only established a new standard of care but also launched a research agenda that has since reshaped central aspects of caring for the mechanically ventilated patients.

The concept of lung protection evolved progressively over this period. Beyond low tidal volumes, driving pressure emerged as a variable independently associated with survival in patients with ARDS, suggesting that normalizing tidal volume to the lung functional size may be as relevant as its absolute value.^(2)^ In parallel, a randomized multicenter trial demonstrated that early and prolonged prone positioning significantly reduced 28- and 90-day mortality in patients with severe ARDS.^(3)^ This evidence consolidated a set of interventions now incorporated into the standard of care for ARDS.^(4)^

More recently, the concept of mechanical power has been proposed by integrating tidal volume, driving pressure, respiratory rate, flow, and PEEP into a single equation.^(5)^ This construct recognizes that ventilator-induced lung injury results not only from a single variable, but from the total mechanical energy transferred to the lung parenchyma per unit of time.^(5)^ Subsequent observational studies have associated higher mechanical power values with increased mortality in critically ill patients, including those without ARDS.^(6)^

If the first part of this 25-year trajectory focused on protecting the lung during the acute phase of mechanical ventilation, the more recent discussion broadens the horizon to what happens after ICU discharge. Post-intensive care syndrome (PICS) describes the set of physical, cognitive, and psychological impairments that may persist in ICU survivors well beyond the hospitalization period.^(7)^ This shift does not replace the acute-phase agenda, but it extends it, incorporating the trajectory of recovery as an integral part of what mechanical ventilation is expected to achieve.

This expansion of focus brings its own tension. Several interventions traditionally used to facilitate lung-protective ventilation, like deep sedation, neuromuscular blockade, and prolonged mechanical ventilation, are also well-established risk factors for ICU-acquired weakness and long-term physical impairment.^(8)^ Protecting the lung and protecting long-term function do not always point in the same direction.

Reconciling these goals has become an active area of clinical and research effort. Early mobilization, even in critically ill patients still receiving invasive ventilation, was first shown to be feasible and to improve functional independence at hospital discharge.^(9)^ The largest trial to date, however, found that higher-intensity mobilization increased adverse events without improving outcomes — a reminder that protecting long-term function is not simply a matter of doing more, but of doing it at the right dose and the right time for each patient.^(10,11)^

Beyond the peripheral musculature, physical function also depends on the respiratory muscles. We now know that it is not only the lungs we must protect during mechanical ventilation, but also the diaphragm. In this context, diaphragm ultrasound has added precision to this effort, enabling real-time titration toward diaphragm-protective ventilation that avoids both the excessive and the insufficient respiratory effort known to injure the diaphragm.^(12)^ These strategies, however, rarely act in isolation. Multicomponent care bundles integrating these elements alongside delirium monitoring and family engagement, such as the ABCDEF bundle, have been associated with reduced mortality, delirium, and long-term disability in critically ill patients,^(13)^ and and post-ICU follow-up clinics increasingly extend this protective intent beyond discharge.^(14)^

Twenty-five years after ARDSNet, the future may no longer be defined solely by how effectively we support patients during mechanical ventilation, but by how successfully our therapeutic strategies in the ICU help survivors return to the lives they led before critical illness.
